# The implementation of physical activity policies in the Netherlands: a study applying the Physical Activity Environment Policy Index (PA-EPI)

**DOI:** 10.1186/s12961-025-01340-w

**Published:** 2025-05-19

**Authors:** Fleur Heuvelman, Jeroen Lakerveld, Kevin Volf, Catherine B. Woods, Suzanne van Mourik-Boelema, Saskia van den Berg, Nicolette R. den Braver

**Affiliations:** 1 Department of Epidemiology and Data Science, Amsterdam UMC, location Vrije Universiteit, Amsterdam, The Netherlands; 2 Department of Physical Education and Sport Sciences, University of Limerick, Limerick, Ireland; 3 Centre for Prevention, Lifestyle and Health, National Institute for Public Health and the Environment, Bilthoven, The Netherlands; 4 Physical Activity for Health Research Centre, Health Research Institute, University of Limerick, Limerick, Ireland; 5 Health Behaviours and Chronic Diseases, Amsterdam, The Netherlands; 6 Upstream Team, Amsterdam UMC, Amsterdam, The Netherlands

## Abstract

**Background:**

Continuing high levels of population physical inactivity necessitate effective government policies to cultivate healthy physical activity (PA) environments. The Physical Activity Environment Policy Index (PA-EPI) is a monitoring framework and tool to assess the implementation of policies that promote PA. This study aims to assess the extent of PA policy implementation in the Netherlands and identify recommendations for action to improve its PA environment, using the PA-EPI.

**Methods:**

The PA-EPI application was a stepwise process in which evidence of policy implementation was collected and validated by government officials. A cross-sectoral coalition of non-government independent experts then rated the extent of implementation of 45 indicators of ideal good practice, comparing them against international best practice. On the basis of these expert ratings, a scorecard categorized indicators into high, medium, low or none/very little implementation. In turn, future implementation recommendations were identified by independent experts, prioritized and disseminated.

**Results:**

The evidence validation by government officials (*N* = 15) yielded minor changes. Independent experts (*N* = 14) gave 10 out of the 45 indicators a low implementation score, 28 a medium score and 7 a high score. The policy domain of transport and the infrastructure support domain of monitoring and intelligence received high implementation scores. The policy domains of mass media and workplace and the infrastructure support domains of leadership, funding and resources and workforce development received only low scores. Some domains received both high and low implementation scores (i.e. the policy domains of education and sport and recreation for all and the infrastructure support domain of governance). A total of 36 policy recommendations and 26 infrastructure support recommendations were identified. The top prioritized policy recommendations fell within the urban design, education, transport, and sport and recreation for all domains. For infrastructure support, the top prioritized recommendations related to the leadership, funding and resources, and governance domains.

**Conclusions:**

The results reveal important policy implementation gaps and strengths across several domains in the Netherlands. Prioritized recommendations are provided for the government to address these implementation gaps and monitor policy change.

**Supplementary Information:**

The online version contains supplementary material available at 10.1186/s12961-025-01340-w.

## Introduction

Physical activity (PA) reduces the risk of developing non-communicable diseases, including type 2 diabetes and coronary heart disease [1–3]. PA also exerts a positive influence on mental health, wellbeing and the environment [4–6]. Despite these known benefits, global levels of PA have declined since 2000 [7]. If this downward trend continues, the global target of a 15% relative reduction in insufficient PA between 2010 and 2030 will not be met [8]. Individual-level interventions to modify PA behaviours yield insufficient effects at the population level because targeting only a single aspect of a complex public health problem, such as PA, is unlikely to achieve the desired population-level outcomes [9, 10].

To effectively address this, a whole-system approach is advised [8, 9, 11, 12]. This approach requires system-level actions embedded within the broader “PA environment”, the latter defined as the collective physical, economic, political and sociocultural contexts, opportunities and conditions that shape one’s PA choices and behaviours [11, 13]. A system encompasses the interactions of its components and the actors involved, requiring different sectors – such as schools, communities, transport, urban design, healthcare, workplaces, mass media and sport and recreation – to acknowledge the importance of PA and collaborate for successfully promoting it [11, 14–16].

Public policy plays a crucial role in driving system-level changes by creating supportive contexts and conditions that facilitate PA [17]. By addressing these “upstream” determinants, that is, the overarching factors that impact health beyond the individual level, governments can generate a shift in population PA levels [18–20]. On the basis of lessons from regulating the alcohol and tobacco industries, regulatory and economic/fiscal policy instruments (e.g. laws, regulations and taxes) are found particularly effective in changing health behaviours [21, 22]. These types of “harder” instruments either compel compliance from those governed (i.e. hardest instruments) or make certain actions easier or more difficult to undertake (i.e. medium–hard instruments) [23]. However, in the PA field, predominantly “soft” policy instruments such as recommendations are provided by the WHO [23]. These soft instruments encourage or nudge individuals towards specific behaviours. To drive more significant improvements in PA behaviour, it might be effective to implement “harder” policy instruments alongside these softer approaches. For example, laws to mandate regular physical education lessons in schools and regulations targeting the built environment/active transport to promote walking and cycling [24].

The use of such “harder” policy instruments could increase the likelihood of achieving policy objectives by facilitating the translation of policy decisions into practice. This iterative process of policymaking, where decisions are enacted and translated into practice, is known as “policy implementation” [25]. Previous research has shown that the implementation of PA policies is suboptimal [26]. Effectively implementing policies is a complex endeavour due to challenges such as ensuring collaboration with various stakeholders and setting realistic goals [27, 28]. Hence, whilst it is crucial for PA policies to exist, it is equally important to gain an understanding of the level of policy implementation, considering these challenges. However, evaluations of implementation are currently lacking.

The Physical Activity Environment Policy Index (PA-EPI) is a tool developed as part of the Policy Evaluation Network (PEN) project, which ran from 2018 to 2023 [17, 29]. It fills this gap by assessing the extent of policy implementation through a system approach [13]. The PA-EPI builds on the Healthy Food Environment Policy Index (Food-EPI), developed by the International Network for Food and Obesity/non-communicable diseases Research, Monitoring and Action Support (INFORMAS), which has been conducted in more than 50 countries [30, 31]. The Food-EPI Australia has been evaluated as useful for informing and guiding obesity prevention policy [32]. Policymakers used the findings from the Food-EPI to inform policy development, identify gaps and support policy decisions. Moreover, civil society actors leveraged the evidence of government progress and examples of successfully implemented policies to bolster their advocacy efforts. Similar to the Food-EPI, the PA-EPI evaluates and benchmarks PA policy implementation of 45 Good Practice Statements or indicators of ideal good practice across eight policy domains (e.g. education, healthcare and transport) and seven infrastructure support domains (e.g. governance, monitoring and intelligence, and workforce development). The Good Practice Statements were developed through a comprehensive process involving policy document reviews, policy audits, scientific literature synthesis, consultation with academic and policy experts globally and consensus workshops by researchers from the Policy Evaluation Network [13]. For example, within the education domain, Good Practice Statements for which there was evidence of impact address physical education in schools, initiatives to promote and support school-related physical activity, shared use of school facilities with the local community and policies that encourage safe active travel to school. More details regarding the development of the PA-EPI are available in the published manuscript [13]. The policy domains pertain to the settings within which PA takes place and where the government can exert influence to promote PA. The infrastructure support domains underpin effective policy implementation. The PA-EPI framework is designed to capture a wide range of PA-related policies and policy instruments, ranging from voluntary agreements and recommendations (“soft policy instruments”) to regulations and laws (“hard policy instruments”). The PA-EPI process results in the identification of implementation strengths and gaps in national PA policies and potential recommendations for action to address these gaps, as well as enables cross-country comparison/benchmarking amongst countries that applied the PA-EPI. Thus far, Ireland is the only country that has applied the PA-EPI, serving as the pilot country for testing the framework developed by the Policy Evaluation Network project in 2022 [13, 33].

Less than half of the Dutch population (45%) met National PA recommendations in 2023 and from 2020 a slight decrease in people adhering to the PA guidelines is observed [34]. According to predictions made by the National Institute for Public Health and the Environment, only 53% of the total Dutch population will meet the National PA recommendations in 2040 if the current policy situation is sustained [35]. This highlights the need for a comprehensive evaluation of current PA policy implementation, including the policy instruments used to shape the PA environment in the Netherlands. The present study therefore aims to (1) assess the extent of PA policy implementation in the Netherlands and (2) determine and prioritize recommendations to improve the implementation of policies aimed at enhancing the PA environment.

## Methods

### Study design and procedure

**Fig. 1:**
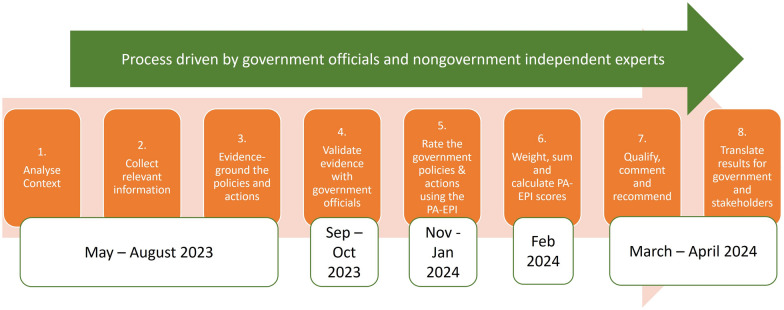
Timeline of the eight step PA-EPI process

This mixed-methods study, conducted between May 2023 and April 2024, utilized questionnaires and focus group discussions to examine the implementation of PA policy in the Netherlands using the PA-EPI. All participants provided informed consent. The Research Ethics Committee of the Amsterdam UMC granted exemption from the requirement for ethical approval. Study protocols were based on those outlined in the PA-EPI publication from Ireland, adapted to the Dutch context (see Fig. 1) [33]. The specific steps and adaptations are described below. More details on the PA-EPI procedure can be found elsewhere (www.jpi-pen.eu). For the definitions of terms related to the evaluation of policy design, implementation and outcomes used in this paper, we refer to the Policy Evaluation Network definitions glossary tool [25].

**Context: The Dutch PA policy landscape**

Public administration in the Netherlands operates at three main levels: the central government, provinces and municipalities [36]. The central government is responsible for national policymaking, whilst provincial authorities implement both national and provincial policies. They oversee areas such as spatial planning in rural regions and regional economic policy. Serving as the “middle level” of public administration, provincial authorities acts as a link between the central government and municipalities [37]. Municipalities implement both national and provincial policies at the local level. The central government comprises ministries, executive agencies, inspectorates, High Councils of State and advisory bodies [38]. Ministries are responsible for a particular policy area. Executive agencies fall under the responsibility of the ministries and include departmental agencies and autonomous administrative authorities, whilst advisory bodies provide policy recommendations to the government. In this study, policy refers to actions taken by the central government, specifically by ministries or their executive agencies, and reflects national public policies. These policy actions may be outlined in broad strategies, action plans, official guidelines, notifications, calls to action, legislation or regulations. A policy action may be documented in a dedicated report or integrated into a broader report covering multiple actions [25].

#### Steps one to three: analysing context (1), collecting relevant information to generate an Evidence Document of implementation of policies and policy actions against the PA-EPI Good Practice Statements (2) and evidence-grounding of the policies and actions (3)

The applicability of all Good Practice Statements was analysed to assess whether they were relevant for the Dutch context. Thereafter, evidence was collected on the extent of implementation of PA policies at the national level, including policy actions, against the 45 PA-EPI Good Practice Statements to create an Evidence Document. First, the Dutch WHO health enhancing physical activity policy audit tool was used as a basis for identifying main policy documents related to PA (such as the PA-specific National Sports Agreement and broader frameworks such as the National Prevention Agreement) and agencies that play an important role in policymaking, informing and implementing (for example, the National Institute for Public Health and the Environment, Knowledge Centre for Sport & PA Netherlands and Mulier Institute) [39]. As the available Dutch health enhancing physical activity policy audit tool document was somewhat outdated (2019), updated policy documents were searched using names of key past policy documents. Secondly, government websites were searched for information and links to additional useful documents. Simultaneously, internet searches based on already identified documents were conducted by extensive snowballing. This involved reference checks of the identified documents, along with searching for initiatives outlined in these policy documents (on both government and nongovernmental websites) and searching for titles of documents to find related action- or implementation plans, policy letters and evaluation or monitoring reports. Lastly, PA-related laws and regulations were searched.

Only national government policies were included in the evidence document. As a result, subnational public policies (e.g. those from municipalities) and policies from nongovernmental bodies unrelated to public policy were excluded. Evidence was extracted from the identified documents and websites and categorized within the PA-EPI domains. The information in the PA-EPI Evidence Document was then summarized by FH, structuring it under the most relevant indicators. Consequently, only content from broader policy documents that directly corresponded to these indicators was included. To ensure accuracy and consistency, the finalized evidence for each indicator was cross-checked by another author (N.R.d.B. or J.L.).

#### Step four: validating evidence with government officials

**Fig. 2:**
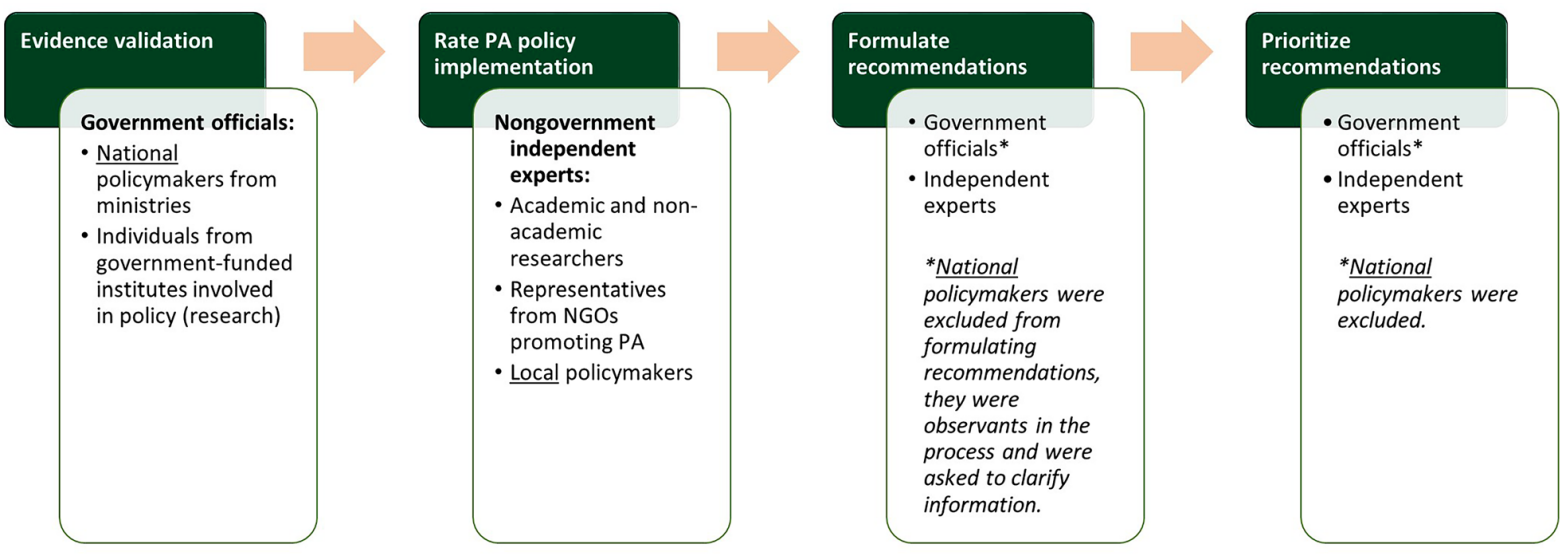
Participants in the PA-EPI process across different phases of data collection

**Fig. 3:**
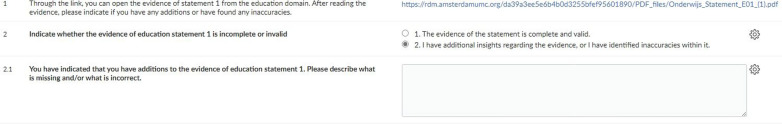
Example from questionnaire sent to government officials

A group of Dutch government officials was assembled to verify the completeness and accuracy of the Evidence Document. Inclusion criteria for these officials were: (1) involvement in national policy and (2) employment at a national government-funded institute. This group was selected on the basis of the research team’s knowledge, consultations with the Dutch Health Enhancing Physical Activity focal point, publicly available information (step two) and the use of a snowball sampling method within this network. It included (1) policymakers from ministries (e.g. Ministry of Health, Welfare and Sport, referred to as Ministry of Health, and Ministry of Infrastructure and Water Management) and (2) professionals working at executive agencies from the ministries (e.g. National Institute for Public Health and the Environment and Knowledge Centre for Sport & PA). We also included professionals from government-funded research institutes who inform policy, due to their close collaboration with policymakers and their in-depth knowledge of national PA policies. For clarity and readability, we will also refer to these individuals as government officials. As a result, this group of government officials represents individuals with extensive knowledge of the Dutch policy system related to PA. Figure 2 provides a detailed overview of the participants at each data collection phase in the PA-EPI process. If a government official agreed to participate, a link to a questionnaire for validating the Evidence Document was sent to them. For each of the Good Practice Statements, government officials indicated whether the evidence was complete or valid (See Fig. 3 for translated questionnaire items). Government officials only validated the Good Practice Statements within their specific fields of expertise. If the evidence was not complete and/or valid, government officials were asked to elaborate on the missing or incorrect aspects, which was reviewed by the core research team (F.H., J.L. and N.R.d.B.). Improvements to the Evidence Document mainly focussed on identifying additional PA policy documents, reorganizing policies and policy actions across indicators, and clarifying the definitions and boundaries of Dutch PA policy. The feedback did not include changes to the evidence regarding policy implementation (e.g. evidence from surveillance reports).

#### Steps five and six: rating the implementation of government policies and actions using the PA-EPI, and weight, sum and calculate rating scores

The extent of implementation of each Good Practice Statement was assessed by relevant Dutch nongovernment independent experts, hereafter referred to as “independent experts”. Inclusion criteria for these experts were (1) independence from the national policymaking process (to objectively rate national policy implementation) and (2) expertise on PA promotion. Their independence from the government was important in minimizing the risk of bias in reporting. This group was selected on the basis of the research team’s knowledge, their published work on PA research within any of the PA-EPI domains, their roles as PA promoters in the Netherlands and the use of a snowball sampling method within this network. This group consequently consisted of (1) academic and nonacademic researchers (i.e. researchers from universities of applied sciences and research universities), (2) representatives from nongovernmental organizations promoting PA (e.g. health foundations) and (3) policymakers from local governments responsible for implementing national PA related policy (Fig. 2). These independent experts received an implementation evaluation questionnaire including the validated Evidence Document and independently rated their perception of the extent of implementation of each Good Practice Statement of the PA-EPI in the Netherlands in comparison with international best practice. As part of the PA-EPI development, policies with outstanding implementation quality were identified and compiled as international best practice exemplars. These exemplars were then incorporated into the Dutch process and provided to experts as a reference. For further details on how these examples were established, we refer to the Irish PA-EPI study [33]. The experts could choose between the following answer options (five-point Likert scale): (1) none/very little implementation (< 20% implemented), (2) low implementation (20–40% implemented), (3) medium implementation (40–60% implemented), (4) high implementation (60–80% implemented), (5) very high implementation (> 80% implemented) and (6) cannot rate. A comment box encouraged independent experts to add recommendations for policy actions and infrastructure support actions to improve the implementation score, these suggestions formed the basis for the step seven.

After collecting independent experts scores, a median rating of the five-point Likert scale was calculated for each Good Practice Statement. The median was chosen because it handles ordinal data better compared with the mean. The median ratings were converted to four categories: (1) very little/no implementation (median ≤ 1.25), (2) low implementation (median > 1.25 to ≤ 2.5), (3) medium implementation (median > 2.5 to ≤ 3.75) and (4) high implementation (median > 3.75). The interrater reliability (Gwet’s AC2 coefficient) was calculated for the implementation ratings using the irrCAC package in R (version 4.4.1).

#### Step seven: qualify, comment and recommend

A full day in-person workshop was organized to formulate recommendations for policy and infrastructure support implementation actions. The following people were invited to the workshop: (1) government officials and independent experts who completed the Evidence Document validation or implementation evaluation questionnaire, (2) government officials and independent experts who did not complete the questionnaires and (3) colleagues of the invited government officials and independent experts to fill in for those who were unable to attend (Fig. 2). In advance of the workshop, participants were provided with the PA-EPI median rating scores and the full list of implementation recommendations from the independent experts. During the workshop, participants were divided into two groups, ensuring balanced expertise in the PA-EPI domains and equal representation from both government officials and independent experts. Each group was guided by an experienced facilitator, whilst a note-taker documented the discussions. For each domain, the groups discussed whether (1) the recommendations formulated in step five could be refined and (2) whether additional recommendations were missing. In an observational role, the national policymakers were instructed to help clarify pertinent information and refine the recommended actions and had no role in formulating new recommendations to maintain independence. At the end of each domain discussion, both groups were asked to present their reformulated or new recommendations to the whole group. A note-taker documented these recommendations, and audio recordings were made with participants' consent.

Following the workshop, a list of implementation recommendations was compiled and circulated to all attendees for confirmation. Subsequently, all government officials and independent experts who participated in any of the PA-EPI steps, excluding national policymakers, were sent a prioritization questionnaire (Fig. 2). They were asked to select and rank their top five policy recommendations (scoring them from one to five) in terms of importance, achievability and equity (i.e. the effect on socioeconomic inequalities in PA). Supplementary file 1 includes the definitions of the ranking criteria presented to the participants on the basis of those used in earlier research [33, 40]. The same prioritization procedure was followed for the infrastructure support recommendations, except there was no ranking based on equitability, in line with earlier research. As a result, five rankings were required (i.e. three for the policy domains and two for the infrastructure support domains). The scores were inverted (i.e. the top-ranked recommendation from an individual rating received a score of five and the fifth ranked recommendation received a score of one), and then summed (importance and achievability). The five implementation recommendations with the highest summed scores were selected as the “priority” implementation recommendations. For the policy domains, an additional top five for the most equitable policy recommendations was created. The five highest-scoring policy recommendations on equitability were included alongside the top five most important and achievable policy recommendations. As a result, the prioritized policy recommendations may exceed five when equitability is taken into account.

#### Step eight: translate results for government and stakeholders

A comprehensive Dutch PA-EPI report has been produced, detailing the PA-EPI process and the resulting outcomes. The PA-EPI results were disseminated at a national event, where key stakeholders such as national policymakers, researchers and PA promoters from nongovernmental organizations were present. During the event, a workshop was conducted to deliberate on the necessary recommendations and identify potential barriers and opportunities. Prior to the event, the final report was distributed to all stakeholders involved in the PA-EPI process via email. Additionally, press statements were issued by our institute and we encouraged relevant parties to do the same.

## Results

### Analysing context

All 45 Good Practice Statements were deemed relevant for the Dutch context.

### Evidence document

Step four (validating evidence with government officials) resulted in an Evidence Document including information on PA policy, policy actions and the implementation of PA policies in the Netherlands. A total of 68 government officials were invited, 23 agreed to validate the evidence and eventually 15 government officials, including 3 national policymakers, completed the full Evidence Document validation questionnaire (Supplementary file 2, Fig. 8). Government officials with expertise in all domains, except mass media, were represented. A government official with specific knowledge in both mass media and health, including PA, could not be identified. The Evidence Document is published online [Reports—JPI PEN (jpi-pen.eu)].

### Level of implementation of PA policy in the Netherlands

For step five, 30 out of 76 (39%) invited independent experts agreed to rate the implementation of the Good Practice Statements. Those who declined participation cited reasons such as time constraints or lack of expertise. A total of 14 (47%) independent experts completed the implementation evaluation questionnaire (Supplementary file 2, Fig. 9).

**Fig. 4:**
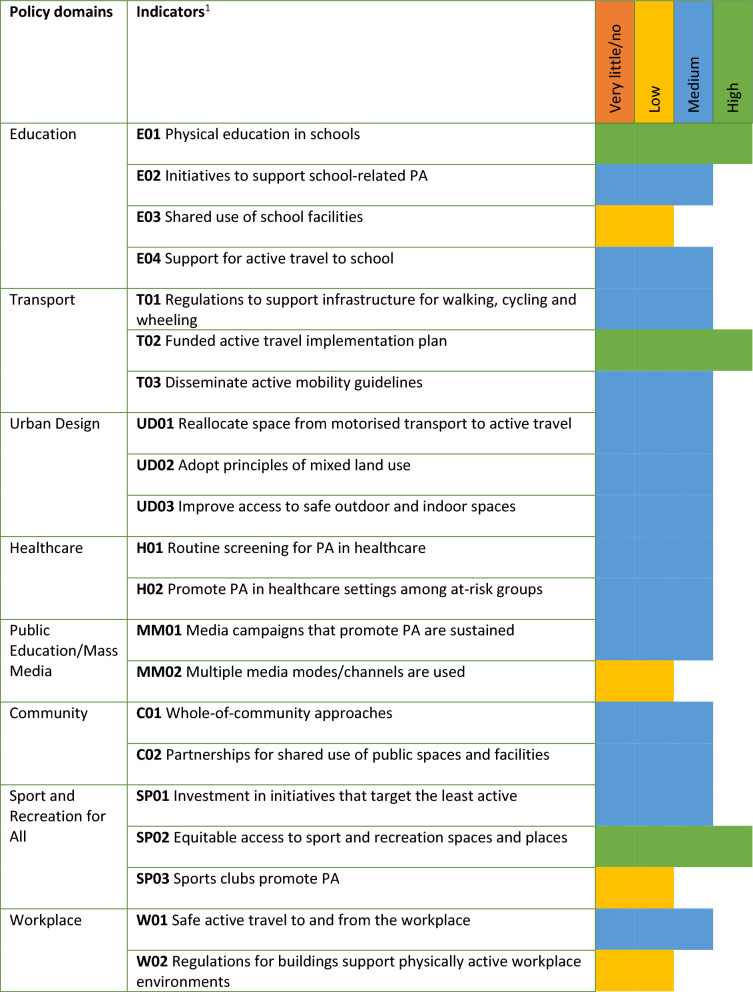
Results of the implementation rating for the policy domains of the PA-EPI. ^1^This is a brief description of the indicators. The full description can be found in the paper on the development of the PA-EPI [13]

The interrater reliability for the implementation ratings was 0.575 (95% CI 0.505–0.645; percentage agreement 85%). For the policy domains, none of the 21 Good Practice Statements received a very little/zero implementation score, 4 Good Practice Statements (19%) received a low implementation score, 14 Good Practice Statements (67%) received a medium implementation score and 3 Good Practice Statements (14%) received a high implementation score (see Fig. 4). The lowest scores emerged in the domains: education, mass media, sport and recreation for all and workplace. Two out of three Good Practice Statements with a high implementation score were also found in the education and sport and recreation for all domains. Moreover, a high implementation score was observed within the transport domain.

**Fig. 5:**
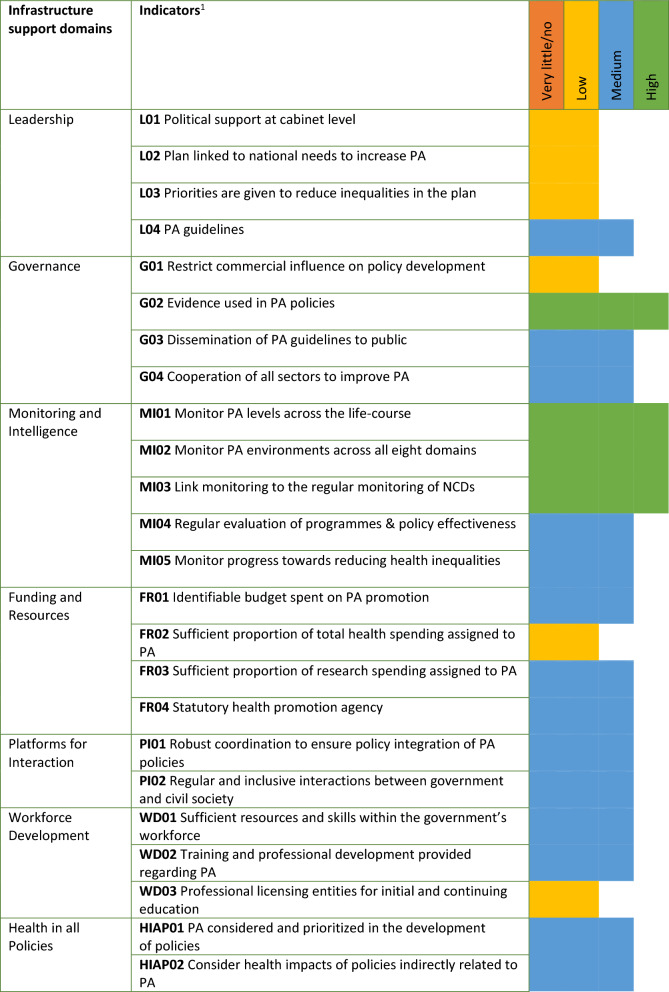
Results of the implementation rating for the infrastructure support domains of the PA-EPI. ^1^This is a brief description of the indicators. The full description can be found in the paper on the development of the PA-EPI [13]

For the infrastructure support domains, none of the 24 Good Practice Statements received a very little/zero implementation score, 6 Good Practice Statements (25%) received a low implementation score, 14 Good Practice Statements (58%) received a medium implementation score and 4 Good Practice Statements (17%) received a high implementation score (See Fig. 5). The low scores emerged in the following domains: leadership, governance, funding and resources and workforce development. Three out of four Good Practice Statements of the leadership domain had a low implementation score. Three out of five Good Practice Statements of the monitoring domain had a high implementation score. In the governance domain both a low and high implementation score emerged.

### Implementation recommendations

In total, 16 participants attended the workshop: 7 government officials, including 1 national policymaker, and 9 independent experts (see Supplementary file 2, Fig. 8 and Fig. 9). The workshop resulted in 62 recommendations, comprising 36 recommendations for policy and 26 for infrastructure support. An overview of all recommendations is presented in Supplementary file 3.

A total of 22 participants ranked the recommendations after the workshop. Amongst them, 14 participants completed the ranking process (i.e. conducted the ranking 5 times), whilst 8 participants did not finish the process by conducting the ranking only 4 times or fewer. This process resulted in 13 most important, achievable and for the policy domains, equitable implementation recommendations. Two of the top five policy recommendations based on a combination of importance and achievability also appear in the top five most equitable policy recommendations, resulting in eight high priority recommendations for policy (Table 1, eight recommendations for the policy domains and five recommendations for the infrastructure support domains).

**Table 1:** Highest ranked implementation recommendations to improve the healthy PA environment for both the policy and infrastructure support domains

Policy domains	
Domain	PA-EPI recommendations for policy action
Urban design^a^	1. Integrate PA into the Environment Act and establish clear conditions or frameworks for incorporating PA aspects (such as space for active transport, play, exercise, sports and mixed land use) into environmental visions, programs and plans that municipalities must comply with. To achieve this, it is important to establish a dedicated position within local government to monitor the integration of PA into spatial planning. This position should also safeguard, monitor and provide feedback (by complying with the national frameworks)
Education	2. Make physical education even less optional and ensure that the education inspectorate structurally assesses its quality on the basis of a set of core indicators
Urban design	3. Formulate clear guidelines for urban design aimed at attractive and socially safe walking and cycling routes through the neighbourhood, near schools, in industrial areas, to recreational areas and across business parks. The following peripheral issues are important: a. Sufficient lighting along the pedestrian and cycle paths; b. Pedestrian and cycle paths along houses (in other words, within sight of residents); c. Prioritize neighbourhoods with low PA levels (where vulnerable groups live who engage in minimal PA)
Transport	4. Formulate guidelines or clear rules in which the STOMP principle^b^ is central to promote active transport. (Local) Policymakers must take these guidelines/rules into account when constructing new neighbourhoods and redeveloping existing neighbourhoods. CROW, a Dutch independent knowledge centre for infrastructure, public space and traffic and transport, can play a role in this guideline development. These guidelines pay attention to: a. Car-free policy, including (limiting) the number of parking spaces in neighbourhoods; b. Number of cycle paths (including *doorfietsroutes*, which are spacious and comfortable bike paths that connect urban regions); c. Amount of high-quality, socially safe and accessible walking paths
Education^a^	5. Increase schools’ obligation to participate in lifestyle programs in which PA plays a role, such as the Healthy School (Gezonde School) or the Healthy Primary School of the Future (Gezonde Basisschool van de Toekomst). As a precondition, the administrative work for these programs, such as the application process, must be simplified for schools. This is especially important for schools that already face many (administrative) burdens
Sport and Recreation for All^a^	6. Implement measures to ensure that financial PA schemes (subsidies) can be used by and are accessible to as many vulnerable groups as possible. For example: a. Increase awareness of the financial schemes; b. Reduce the restrictions of the regulations that limit access; c. Ensure uniform regulations across all municipalities (in other words, expand the regulations in specific municipalities); d. Reduce the administrative burden for applying for schemes (in other words, simplify the application procedure). For example, pay attention to groups facing language barriers who wish to utilize these schemes
Sport and Recreation for All^a^	7. Facilitate co-creation of national PA initiatives targeting the least active and vulnerable groups, involving stakeholders (including at least representatives of the target group), and considering structural barriers for PA (such as poverty and stress). The goal is to integrate PA as a structural component of a broader strategy to address challenges faced by these groups. For example, PA can be included as a potential intervention within poverty policy. It is important for the PA sector to remain flexible and recognize that PA may not always be a high priority for these groups
Urban design^a^	8. Establish requirements for municipalities to ensure universal accessibility of public spaces, guaranteeing accessibility for all users (including pedestrians). Consider the following requirements: a. A bench must be available every x meters; b. There must be x public toilets in a neighbourhood; c. x playgrounds must be designed according to the *samenspeelnorm*: 100 – a playground where everyone (100%) is welcome; 70 – a playground that is at least for 70% accessible to everyone; 50 – a playground where at least 50% of the playground equipment is playable for every child and is aimed at meeting and playing together

Infrastructure support domains	
Domain	PA-EPI recommendations for infrastructure support action
Leadership	1. Implement structural PA policy (and associated PA goals) that extends beyond a usual 4-year government term, ensuring long-term commitment
Funding and resources	2. Increase funding for prevention initiatives, with a significant focus on cross-domain PA
Leadership	3. Develop PA guidelines (potentially on the basis of the WHO PA guidelines) tailored for vulnerable groups (such as the chronically ill, pregnant women and people with disabilities), to complement the guidance provided by Knowledge Centre for Sport & PA
Governance	4. Promote/facilitate the implementation of toolboxes and toolkits developed by Knowledge Centre for Sport & PA and the National Institute for Public Health and the Environment, aimed at securing and utilizing knowledge (including elements of proven effective interventions) in the development of (local) PA policy. This will aid in the effective use of available knowledge a. As a condition for subsidies for scientific research, require collaboration with a semi-governmental organization (such as Knowledge Centre for Sport & PA or the National Institute for Public Health and the Environment) to ensure practical translation
Governance	5. Launch media campaigns and additional communication strategies to increase awareness and the importance of PA, with a specific focus on the PA guidelines, amongst the general population

^a^The five most equitable policy recommendations are combined with the most important and achievable policy recommendations, resulting in a total of eight prioritized policy recommendations

^b^With STOMP, priority is given to sustainable modes of transport in the design process, whilst less priority is given to less sustainable modes. The central order of priority is as follows: walking, cycling, public transport, mobility as a service and personal transport

**Fig. 6:**
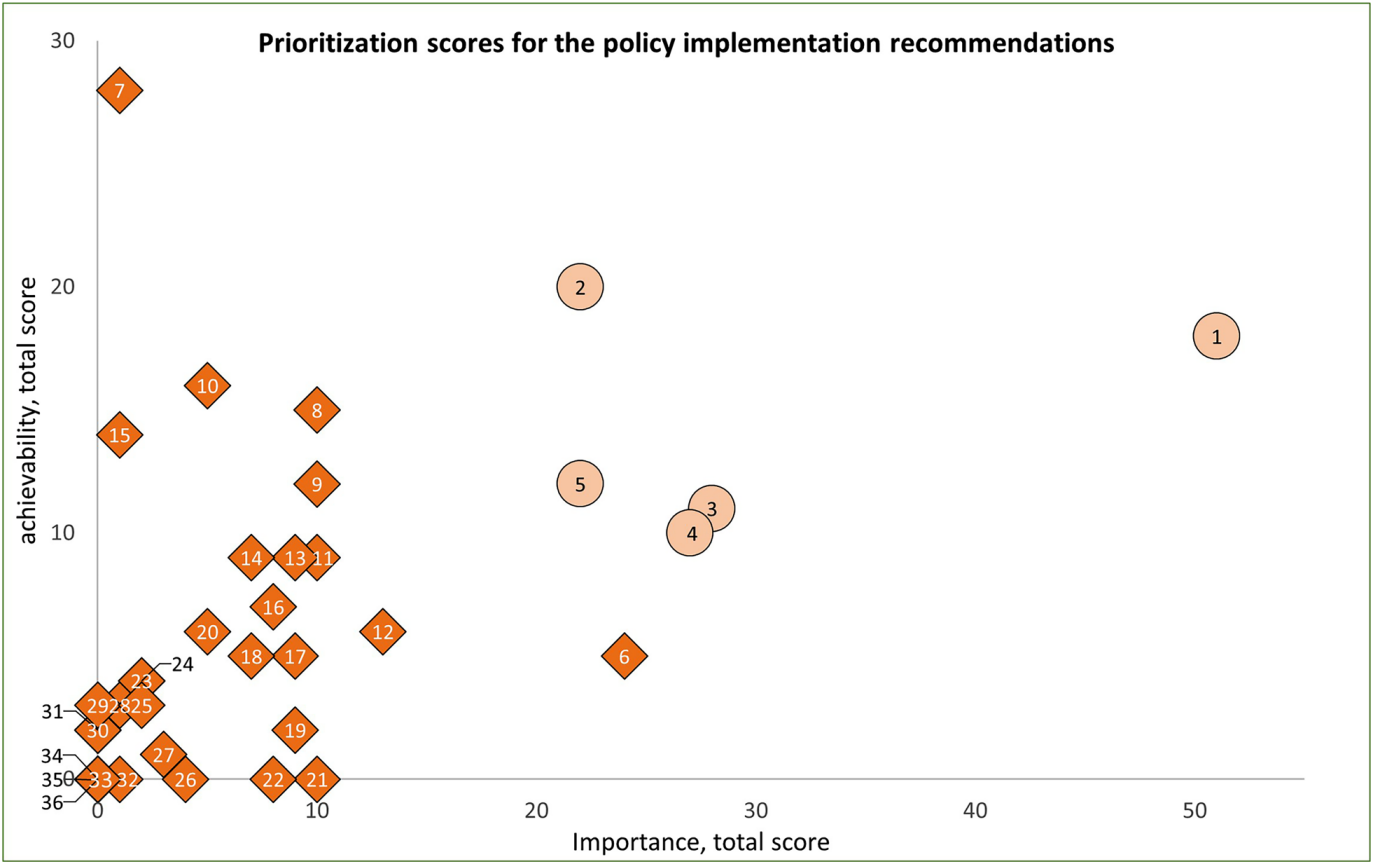
Prioritization of the 36 recommendations for the policy domains. The numbers represent each recommendation. The five highest-priority recommendations, based on importance and achievability, are displayed in light orange circles

**Fig. 7:**
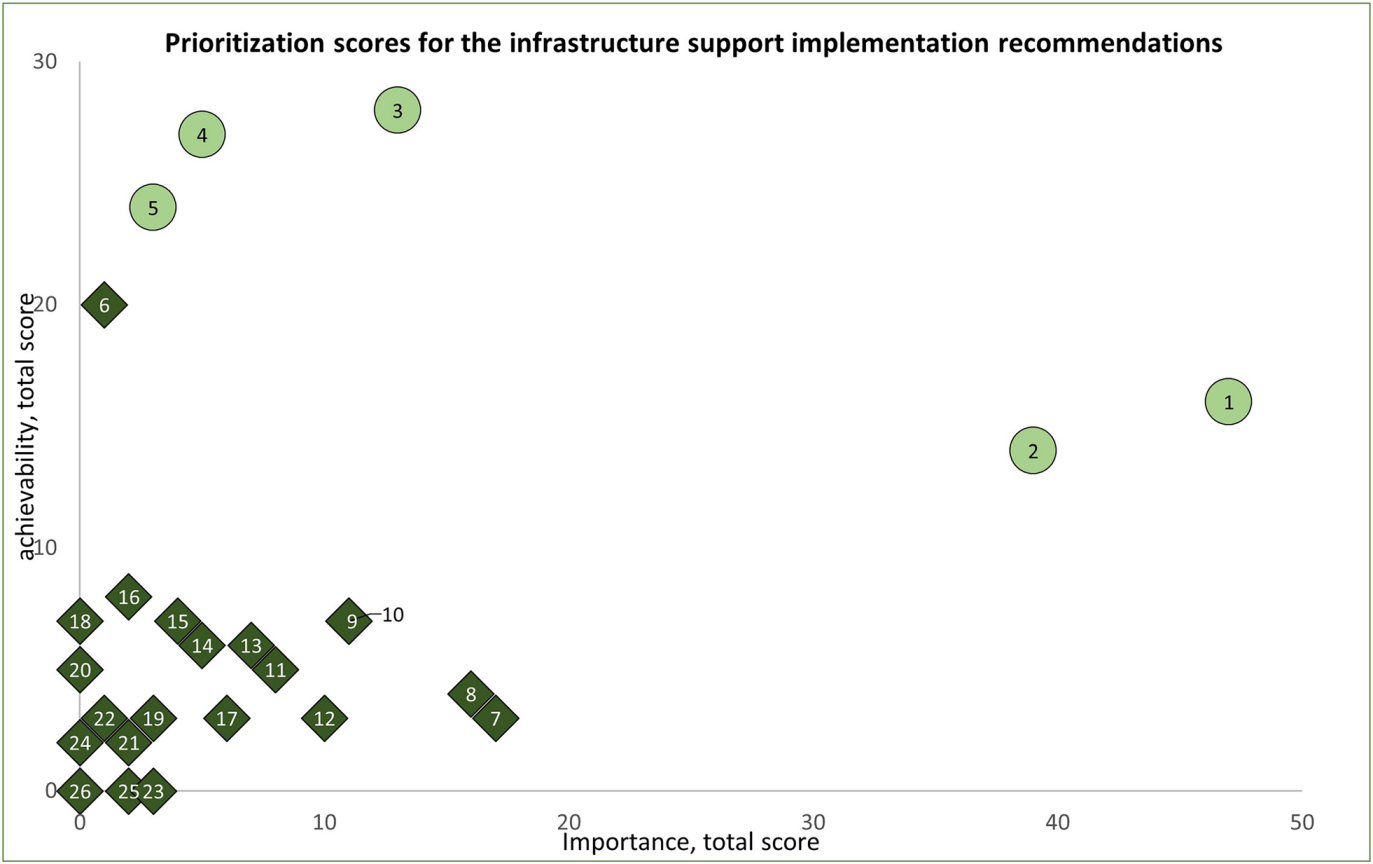
Prioritization of 26 recommendations for the infrastructure support domains. The numbers represent each recommendation. The five highest-priority recommendations, based on importance and achievability, are displayed in light green circles

The total scores based on importance and achievability of all 62 recommendations are displayed in Figs. 6 and 7. Two of the top five infrastructure support recommendations have a summed importance score of 39 or higher. All other infrastructure support recommendations have a summed importance score of 17 or lower, indicating that recommendations three, four and five (Table 1) are perceived as highly achievable but relatively unimportant compared with other recommendations.

### Dutch PA-EPI report and dissemination

The Dutch PA-EPI report was published online [Reports—JPI PEN (jpi-pen.eu)] and presented to policymakers and other key stakeholders. The official launch took place on 1 July 2024, during an event organized by the Movement Alliance (Beweegalliantie), a national network established by the Ministry of Health to overcome obstacles and stimulate innovative PA initiatives, encouraging more Dutch citizens to become physically active. At the event, the report was formally presented to the Director of Public Health at the Ministry of Health. Potential barriers and opportunities of the recommendations were identified during the workshop. The report was widely disseminated to relevant stakeholders. Press releases were issued by both our institute and other organizations involved in promoting PA.

## Discussion

The PA-EPI tool was applied to assess the level of implementation of Dutch policies relating to the PA environment. A comprehensive overview of current national PA policies, actions and policy implementation was captured in an Evidence Document, which was then thoroughly reviewed by Dutch independent experts such as academics and PA promoters from nongovernmental organizations. The indicators of ideal good practice received a range of implementation scores, highlighting both gaps and strengths in the implementation of PA policies in the Netherlands. A total of 13 prioritized implementation recommendations were identified aiming to create healthier PA environments in the Netherlands. The process of applying the PA-EPI facilitated coalition building and initiated dialogues on PA across various policy domains with relevant stakeholders.

### Policy implementation scores

A total of seven indicators received high implementation scores in the Netherlands. Some of these can be reasonably explained, such as the high scores of the monitoring and intelligence indicators (MI01, MI02 and MI03). The National Institute for Public Health and the Environment coordinates, on behalf of the Ministry of Health, the annual or bi-annual monitoring of PA indicators, such as PA guideline adherence, percentage of people who engage in sport and the degree to which the living environment facilitates PA [41]. Moreover, the National Institute for Public Health and the Environment is involved in the execution of the Lifestyle Monitor (de Gezondheidsenquête/Leefstijlmonitor), which monitors both PA and non-communicable diseases annually. Secondly, the only high-scoring indicator in the transport domain (T02) might reflect the Netherlands’ internationally recognized leadership in active transport, particularly cycling [42]. This specific transport indicator relates to having a funded active travel implementation plan. The leadership is supported by dense and modern infrastructure, promoting cycling as a primary mode of transportation [43]. However, despite our extensive cycling network, most trips for transport are still done by car [44]. Furthermore, new cycling infrastructure-related challenges have emerged, such as a rise in cycling accidents, which might be due to the high traffic intensity on cycling paths and the increased heterogeneity in bikes, that is, the growing popularity of electric bikes [45]. Therefore, the medium score for another transport indicator (T01: regulations that provide infrastructures to support safe walking, cycling and/or wheeling) indicates that even within our current cycling infrastructure, improvements in the implementation of policies related to traffic may be necessary to address these issues.

The low implementation rating for the workplace indicator (W02) – regulations for buildings support physically active workplace environments – aligns with research showing that Dutch workers spend the most time sitting in Europe [46]. In 2022, they sat for an average of 8.9 h per day. The Vital Company programme (Vitaal Bedrijf), launched in 2020 and supported by two Dutch ministries (Ministry of Health and Ministry of Social Affairs and Employment), aims to promote workplace health, including PA [47]. It offers companies practical tools such as vitality scans and consultations. Companies receive a certificate if they assess employee vitality and offer activities of at least two of the five vitality themes (e.g. PA and nutrition), with at least 25% participation [48]. To date, only 22 companies have received this certificate, a small number compared with the 1.5 million small- and medium-sized companies in the Netherlands [49]. This indicates the limited success of the implementation of such programs, likely due to their “soft” policy nature; they primarily offer recommendations to inform companies [23]. Following the completion of the Dutch Evidence Document used for policy implementation rating, companies could apply for a subsidy from the Ministry of Social Affairs and Employment to enhance sustainable employability [50]. This subsidy, categorized as a “harder” policy instrument (i.e. economic/fiscal policy instruments), may be effective in improving policy implementation.

The Netherlands was the second country to apply the PA-EPI, following Ireland in 2022 [33]. In contrast to the Netherlands, Ireland showed better implementation of the indicator sport clubs promoting PA (SP03). This may be partly attributed to the Gaelic Athletic Association, an amateur sports organization that promotes Indigenous Irish games and sports and has a wide reach across communities in Ireland [51]. Its strong community ties make it an ideal partner for governments aiming to implement health-promoting sports club initiatives. These examples highlight the potential for mutual learning.

### Policy implementation recommendations and prioritization

Findings of this research are consistent with previous efforts and studies calling for more enactment and the implementation of structural, less voluntary and consistent national policy interventions with clearly defined responsibilities [23, 52]. The Netherlands Sports Council, the independent advisory body for the cabinet and parliament that focusses on strengthening the significance of sport in society, concludes in their most recent report that current PA policy predominantly aims to inform individuals and facilitate their choices, whilst there is an absence of structural, regulatory (hard) PA policies [53]. The deficiency in the use of coercive methods appears to be characteristic of the Dutch public health policy landscape, as similar traits have been seen in the food environment [54, 55]. A shift towards “harder” policy instruments (e.g. laws and regulations) to improve PA policy implementation, such as mandating municipalities to integrate PA into their environmental plans and requiring municipalities to prioritize active transport in neighbourhood construction (e.g. by creating car-free zones and expanding safe cycling/walking paths), appears important for creating an environment that not only encourages, but also systematically supports, PA [56]. Frameworks, such as the Regulatory Approaches to Movement, PA, Recreation, Transport and Sport, can support national policymakers to implement legal interventions which support or strengthen a whole-system approach and enhance policy implementation to promote PA [57].

Similar to the findings in Ireland, there was some discrepancy between the highest implementation ratings and the highly prioritized recommendations for action [33]. Some highly prioritized recommendations correspond with indicators that received high implementation ratings, which was unexpected. Several potential explanations could account for this misalignment between scores and recommendations. Firstly, rater expertise and experience could be a contributing factor. A similar approach to the Food-EPI for recruiting independent experts was employed [33]. Unlike food policy, which is centred within the health domain, PA policy is more fragmented across domains and ministries, such as health, transport and education. This fragmented expertise may have resulted in artificially higher ratings in domains outside the independent experts’ areas of expertise, particularly if a large number of policy actions were mistaken for a high level of implementation. To enhance the validity and reliability of the implementation scoring, it is recommended that independent experts rate the policy implementation only within their specific domains of expertise. Secondly, the misalignment could be due to ceiling effects on the rating scale, indicating that the scale might not be sensitive enough to detect small changes. It could be argued that adjusting the scale to allow for more refined levels of scoring would improve its sensitive and accuracy. Finally, it is possible that the implementation was scored highly in comparison with international best practice. However, the independent experts might believe that there is still room for improvement, leading them to make and prioritize recommendations accordingly.

Respondent fatigue may have influenced the formulation of recommendations in step five and the prioritizing of actions in step seven. The first three domains presented in the implementation evaluation questionnaire, education, transport and urban design, received most implementation recommendations. This could be due to the lengthiness of the Evidence Document, leading independent experts to suggest more recommendations for the first domains presented in the implementation evaluation questionnaire. This imbalance was mitigated during the workshop by changing the order of the domains when modifying or formulating recommendations. Moreover, the recommendations for action presented first in the prioritization questionnaire were predominantly prioritized. The large number of recommendations may have also influenced prioritization outcomes in the Irish PA-EPI study [33]. This highlights the importance of carefully managing the number of recommendations resulting from the workshop. However, there is robust evidence supporting PA policies benefitting children in school settings and promoting walking and cycling (i.e. infrastructure policies improving the built environment). This justifies the prioritization of implementation recommendations in the education, urban design and transport domains [24], making recommendations in these areas valid on the basis of the available evidence.

The prioritization of infrastructure support recommendations revealed a notable discrepancy between their perceived importance and achievability. Amongst the five highest prioritized infrastructure support recommendations, only two received high importance scores. The remaining recommendations were included in the top five on the basis of their high achievability. This discrepancy can be reasonably explained by the nature of the recommendations. For instance, developing PA guidelines for vulnerable groups (recommendation three) and launching media campaigns for disseminating PA guidelines (recommendation five) are relatively straightforward to implement but are unlikely to have a significant impact on PA [58]. Therefore, it might be important, especially for the infrastructure support recommendations, to be formulated with careful consideration of both importance and achievability during the workshop to maximize their impact on PA behaviours. Moreover, the scoring range for importance in the Netherlands (0–50) is higher compared with the range in Ireland (0–35), indicating that a few recommendations in the Dutch context were considered highly important by a relatively large number of the participants [33].

### Strengths and limitations

The study has several strengths. First, the whole-systems approach underpinning the PA-EPI is a major strength. PA extends beyond sports and the health sector, involving multiple sectors. This comprehensive tool ensures that all relevant sectors are considered, enhancing PA promotion. Second, the PA-EPI process successfully brought together individuals with diverse expertise and various roles in promoting PA, crucial for aligning PA policy across sectors. This coalition-building component fosters effective cross-domain policy implementation. Moreover, the National Institute for Public Health and the Environment emphasized the importance of continuous, cross-domain and cross-sectoral PA policy in their evaluation of preventive health policy from 2006 to 2018 [59]. Internationally, this has also been acknowledged by the WHO’s Global Action Plan on PA (GAPPA) [8]. The PA-EPI serves as a foundational tool for monitoring and evaluating these integrated policy efforts. Additionally, the compilation of an Evidence Document can be seen as an important strength as it entails concrete examples of good practice in PA policy and its implementation. As a result, the PA-EPI provides a mechanism for countries to understand their current implementation status in relation to their PA policy, set actionable goals and establish pathways to achieve these goals. It also offers a method for documenting progress. Lastly, the PA-EPI enables cross-country comparisons once applied in multiple countries, promoting mutual learning and refinement of the PA-EPI process, making it applicable across European countries.

There are also some limitations to this study. First, the absence of mass media expertise in the development of the Evidence Document may have influenced the quality of the evidence. Mass media is not a stand-alone policy setting, but is integrated within other settings such as health and transport, which could explain the absence of expertise. Although the evidence is based on policy documents and government websites, which supports its validity, we cannot entirely rule out the possibility that some relevant information may be missing. Second, as previously explained, the implementation scoring may present an optimistic view of the actual implementation of PA policies. Moreover, participating in the PA-EPI process is a labour-intensive task, particularly for the independent experts, which might have influenced response rates. However, this burden can be alleviated by focussing on rating the implementation of indicators that align with the experts’ fields of expertise. Another potential limitation of this study is that the policy implementation scoring system was somewhat subjective and may be interpreted differently by experts, highlighting the need for transparency, and clear guidance on how to perform the ratings. This variability in interpretation is reflected in the interrater reliability score, which indicates moderate agreement. Finally, to enhance the validity of the results, it is important to include individuals with diverse PA expertise throughout the various PA-EPI steps. Whilst individuals with a range of expertise were successfully included, with the exception of mass media, some domains may still have been underrepresented or overrepresented. Nonetheless, the formulated and prioritized recommendations remain highly relevant, even when they are somewhat clustered within certain domains.

### Suggestions for future research

This study produced scorecards evaluating the implementation of national government PA policies and provided recommendations to improve the Dutch PA environment. These scorecards can serve as a foundation for monitoring the progress of policy implementation over time, ideally every 4–5 years. Matching these scorecards and the recommendations for action to policy letters and/or surveillance reports over time will enable monitoring of progress on the implementation of the recommendations. This approach not only aids in evaluating our national PA policy implementation, but also contributes to a global database for monitoring and evaluating the implementation of European PA policies. To enhance the utility of this study and Ireland’s PA-EPI study, it is recommended that the PA-EPI be applied in multiple countries and eventually compare the results. To enhance the applicability of the recommendations and facilitate these cross-country comparisons, standardized criteria and guidance for rating policy implementation and formulating policy recommendations should be established. These criteria should include elements such as timelines, resource allocation and leadership roles. Finally, vulnerable groups, such as socioeconomically disadvantaged people, migrants and refugees and people with disabilities, are less likely to meet PA guidelines [60–63]. PA policies aimed at the general population, without addressing disparities, might inadvertently increase health inequities [64]. Integrating an equity perspective throughout the PA-EPI process, and formulating policy recommendations that consider these inequities, is therefore advised.

## Conclusions

This study shows that some PA policies in the Netherlands have high implementation; especially in the policy domain of transport and in the infrastructure support domain of monitoring. However, low levels of implementation were found in the policy domains of mass media and workplace and in infrastructure support domains of leadership, funding and resources and workforce development. In some domains high and low implementation was observed (i.e. the policy domains of education and sport and recreation for all and the infrastructure support domain of governance). Policy recommendations for addressing implementation gaps are provided and include prioritizing of PA in urban design—for example, by making PA part of the Environment and Planning Act—making physical education even less optional, emphasizing active transport in local government guidelines, ensuring participation of schools in lifestyle programs, increasing accessibility of financial PA schemes, co-creating PA initiatives with inactive groups and ensuring universal accessibility of public spaces. Recommendations for infrastructure support involve implementing PA policy that extends government terms, increasing funding for PA promotion, developing PA guidelines for vulnerable groups, using existing knowledge more efficiently in PA policymaking and developing media campaigns for disseminating PA guidelines. The top-priority recommendations highlight the need for integrated collaboration across policy areas and ministries.

## Supplementary Information

Supplementary material 1.

Supplementary material 2.

Supplementary material 3.

## Data Availability

The datasets used and/or analysed during the current study are available from the corresponding author on reasonable request.
