# Supplementary material for: The implementation of physical activity policies in the Netherlands: a study applying the Physical Activity Environment Policy Index (PA-EPI)
Source: Health Res Policy Syst. 2025 May 19;23:59. doi: 10.1186/s12961-025-01340-w (PMC12090461; doi:10.1186/s12961-025-01340-w)
Supplement: Supplementary file 1 — Supplementary material 1. [file 12961_2025_1340_MOESM1_ESM.docx]

Supplementary file 1: Criteria for prioritizing

**Importance criteria**

| **Need** | **Impact** | **Other positive effects** | **Other negative effects** |
| --- | --- | --- | --- |
| The size of the implementation gap | The effectiveness of the action on improving PA environments (including reach and effect size) | For example, on protecting rights of children | For example: regressive effects on household income, infringement of personal liberties |

**Achievability criteria**

| **Feasibility** | **Acceptability** | **Affordability** | **Efficiency** |
| --- | --- | --- | --- |
| How easy or hard the action is to implement | The level of support from key stakeholders including government, the public, public health and industry | The cost of implementing the action | The cost-effectiveness of the action |

**Equity criteria**

| **Socio-economic effect** | **Structures vs. Individuals** |
| --- | --- |
| Progressive/regressive effects on reducing PA-related inequalities | The extent to which a given policy requires environmental change rather than individual choices |
