# Supplementary material for: The implementation of physical activity policies in the Netherlands: a study applying the Physical Activity Environment Policy Index (PA-EPI)
Source: Health Res Policy Syst. 2025 May 19;23:59. doi: 10.1186/s12961-025-01340-w (PMC12090461; doi:10.1186/s12961-025-01340-w)
Supplement: Supplementary file 2 — Supplementary material 2. [file 12961_2025_1340_MOESM2_ESM.docx]

Supplementary file 2: Flowcharts of government officials and nongovernment independent experts

Reasons for not participating (n = 45)

- No reaction (n = 13)
- Colleague recommended^1^ (n = 14)
- Lack of expertise on topic (n = 7)
- Leave, retired, personal circumstances (n = 3)
- Time constraints (n = 2)
- No reason (n = 1)
- Undelivered mail returned to sender (n = 5)

Invited government officials

n = 68

Government officials agreed to participate

n = 23

Reasons for not completing questionnaire (n = 8)

- No reaction (n = 6)
- Lack of expertise on topic/ questionnaire was too complex (n = 1)
- No informed consent (n = 1)

Government officials that completed questionnaire

n = 15

Independent experts in the PA-EPI workshop^3^

n = 9

Government officials in the PA-EPI workshop^2^

n = 7

*^1^This occurred when a colleague was determined to be more suitable (having more experience on the research topic) compared to the invited official.*

*^2^Workshop participants included government officials who completed the questionnaire, colleagues of contacted officials who were unable to attend the workshop, and government officials within the research team's network who had not been involved in previous PA-EPI steps, but were identified as suitable contributors for the workshop.*

*^3^See Figure 8 for the flowchart of participating nongovernment independent experts*

Figure 7: Flowchart of government officials

Invited independent experts

n = 76

Reasons for not participating (n = 46)

- No reaction (n = 23)
- Colleague recommended^1^ (n = 5)
- Lack of expertise on topic (n = 5)
- Leave, retired, personal circumstances (n = 5)
- Time constraints (n = 6)
- No reason (n = 1)
- Undelivered mail returned to sender (n = 1)

Independent experts agreed to participate

n = 30

Reasons for not completing questionnaire (n = 16)

- No reaction (n = 6)
- Lack of expertise on topic/ questionnaire was too complex (n = 5)
- Leave or personal circumstances (n = 3)
- Time constraints (n = 2)

Independent experts that completed questionnaire

n = 14

Government officials in the PA-EPI workshop^3^

n = 7

Independent experts in the PA-EPI workshop^2^

n = 9

*^1^This occurred when a colleague was determined to be more suitable (having more experience on the research topic) compared to the invited expert.*

*^2^Workshop participants included independent experts who completed the questionnaire, those who did not complete it (e.g., individuals with domain-specific knowledge), colleagues of contacted experts who were unable to attend the workshop, and colleagues of workshop participants who were thought to also contribute significantly.*

*^3^See Figure 7 for the flowchart of participating government officials*

Figure 8: Flowchart of nongovernment independent experts
