# Supplementary material for: The implementation of physical activity policies in the Netherlands: a study applying the Physical Activity Environment Policy Index (PA-EPI)
Source: Health Res Policy Syst. 2025 May 19;23:59. doi: 10.1186/s12961-025-01340-w (PMC12090461; doi:10.1186/s12961-025-01340-w)
Supplement: Supplementary file 3 — Supplementary material 3. [file 12961_2025_1340_MOESM3_ESM.docx]

Supplementary file 3: All 62 policy and infrastructure support recommendations

Implementation recommendations for policy and infrastructure support for creating a healthy PA environment in the Netherlands, recommended by participants. Recommendations are shown in order of ranking on a combination of importance and achievability.

| Recommendations for policy action | | |
| --- | --- | --- |
| No. | Domain | Recommendation |
| 1 | Urban design | Integrate PA into the Environment Act and establish clear conditions or frameworks for incorporating PA aspects (such as space for active transport, play, exercise, sports and mixed land use) into environmental visions, programs and plans that municipalities must comply with. To achieve this, it is important to establish a dedicated position within local government to monitor the integration of PA into spatial planning. This position should also safeguard, monitor and provide feedback (by complying with the national frameworks). |
| 2 | Education | Make physical education even less optional and ensure that the education inspectorate structurally assesses its quality on the basis of a set of core indicators |
| 3 | Urban design | Formulate clear guidelines for urban design aimed at attractive and socially safe walking and cycling routes through the neighbourhood, near schools, in industrial areas, to recreational areas and across business parks. The following peripheral issues are important:   1. Sufficient lighting along the pedestrian and cycle paths; 2. Pedestrian and cycle paths along houses (in other words, within sight of residents); 3. Prioritize neighbourhoods with low PA levels (where vulnerable groups live who engage in minimal PA). |
| 4 | Transport | Formulate guidelines or clear rules in which the STOMP^1^ principle is central to promote active transport. (Local) Policymakers must take these guidelines/rules into account when constructing new neighbourhoods and redeveloping existing neighbourhoods. CROW, a Dutch independent knowledge centre for infrastructure, public space and traffic and transport, can play a role in this guideline development. These guidelines pay attention to:   1. Car-free policy, including (limiting) the number of parking spaces in neighbourhoods; 2. Number of cycle paths (including *doorfietsroutes*, which are spacious and comfortable bike paths that connect urban regions); 3. Amount of high-quality, socially safe and accessible walking paths. |
| 5 | Education | Increase schools' obligation to participate in lifestyle programs in which PA plays a role, such as the Healthy School (Gezonde School) or the Healthy Primary School of the Future (Gezonde Basisschool van de Toekomst). As a precondition, the administrative work for these programs, such as the application process, must be simplified for schools. This is especially important for schools that already face many (administrative) burdens. |
| 6 | Healthcare | Establish a coordinated approach by integrating a structural intermediary, such as a lifestyle counter (Leestijlloket), that facilitates connections between care providers (e.g., GPs, hospitals, and physiotherapists) and PA opportunities, into insured care. This intermediary identifies the patient's needs and offers suitable PA options, and serves as a backup to revise the PA plan if necessary. |
| 7 | Healthcare | Encourage the development and use of training programs that contribute to increasing healthcare professionals' knowledge about PA. |
| 8 | Healthcare | Include physiotherapy and/or effective PA interventions (such as those in the Combined Lifestyle Intervention (GLI)) and/or (short-term) PA initiatives into the basic insurance. |
| 9 | Education | Require municipalities to introduce car-free zones around school areas by using School Streets (Schoolstraten), for example. |
| 10 | Education | Encourage municipalities to consider multifunctional use of space in the planning of new schoolyards by including it in zoning plans, for example. |
| 11 | Transport | Implement regulations to standardize speed limits within urban areas, making 30 km/h the nationwide norm. |
| 12 | Sport and Recreation for All | Implement measures to ensure that financial PA schemes (subsidies) can be used by and are accessible to as many vulnerable groups as possible. For example:   1. Increase awareness of the financial schemes; 2. Reduce the restrictions of the regulations that limit access; 3. Ensure uniform regulations across all municipalities (in other words, expand the regulations in specific municipalities); 4. Reduce the administrative burden for applying for schemes (in other words, simplify the application procedure). For example, pay attention to groups facing language barriers who wish to utilize these schemes. |
| 13 | Transport | Implement pricing measures to discourage private and commuting car use:   1. Increase fuel taxes and driving costs; 2. Raise parking fees, including for permit holders; 3. Make leasing cars more expensive. |
| 14 | Healthcare | Establish procedures to integrate PA guidelines into care standards. These procedures should cover the inclusion of PA guidelines in both existing and newly developed or revised care standards, as well as the integration of updated PA guidelines in care standards. |
| 15 | Education | Provide more education at schools to teachers and pupils on the importance of (early) motor skills and the importance of PA. |
| 16 | Urban design | Encourage municipalities to maintain or develop sufficient facilities and incentives that meet residents' needs for walking, cycling, or rolling (for wheelchair users). This could include shops and employment opportunities within cities. |
| 17 | Education | Ensure the suitability and competence of physical education teachers by, for example, promoting the use of specialized subject teachers, particularly in primary schools. |
| 18 | Urban design | Establish requirements for municipalities to ensure universal accessibility of public spaces, guaranteeing accessibility for all users (including pedestrians). Consider the following requirements:   1. A bench must be available every x meters; 2. There must be x public toilets in a neighbourhood; 3. x playgrounds must be designed according to the *samenspeelnorm*:   100 - a playground where everyone (100%) is welcome;  70 - a playground that is at least for 70% accessible to everyone;  50 - a playground where at least 50% of the playground equipment is playable for every child and is aimed at meeting and playing together. |
| 19 | Transport | Lower prices and improve the quality of public transport. Ensure sufficient capacity, including more frequent service and greater coverage. Measures should address both vulnerable groups who experience limitations in public transport use and the transition from (lease) car users to public transport use. |
| 20 | Community | In collaboration with municipalities, establish national targets (Key Performance Indicators (KPIs)) and conditions for new community initiatives focused on PA (e.g., a new CityDeal). These conditions should include:   1. Structural monitoring and evaluation of community initiatives throughout their duration; 2. **Multidisciplinary collaboration:** involving expertise from at least three different domains for smaller initiatives and as many as possible for larger ones; 3. Stakeholder analysis: identification of potential cooperation partners by the initiative's initiator; 4. Securing ownership of a community initiative by designation of a stakeholder responsible for providing mandatory feedback (including monitoring reports) to an agreed-upon (semi-)government organization. 5. Incorporation of existing proven effective components and practices related to community initiatives. |
| 21 | Workplace | Prioritize prevention, including PA, in the Occupational Health and Safety Act (Arbowet) by, for example, establishing regulations on the duration of sedentary behaviour and physical overload. As physical overload may prevent employees from exercising outside of working hours. |
| 22 | Workplace | Encourage employers to offer financial incentives to promote PA among employees, such as discounts on gym memberships or extra days off for regular PA. Extend these incentives to unemployed individuals as well, potentially through organizations like UWV. |
| 23 | Transport | Facilitate the establishment of Travel Training focused on cycling and public transport use and support the delivery of this training to ethnic minorities, low-literate people, elderly and other relevant groups. |
| 24 | Sport and Recreation for All | Facilitate co-creation of national PA initiatives targeting the least active and vulnerable groups, involving stakeholders (including at least representatives of the target group), and considering structural barriers for PA (such as poverty and stress). The goal is to integrate PA as a structural component of a broader strategy to address challenges faced by these groups. For example, PA can be included as a potential intervention within poverty policy. It is important for the PA sector to remain flexible and recognize that PA may not always be a high priority for these groups. |
| 25 | Education | Ensure the continuity of evidence-based exercise initiatives for schools—primarily short-term programs—from the RIVM's Database for Recognized Interventions, in collaboration with Knowledge Centre for Sport & PA. These initiatives should include sustainable pathways to PA, ensuring a structural and lasting integration of PA. |
| 26 | Transport | Currently, the significant speed differences on cycle lanes—due to varying users like children, e-bikes, speed-pedelecs, and regular cyclists—create conflicts, accidents, and a sense of insecurity. Infrastructure should be revised to better support these different modes of transport:   1. Designate specific sections of the highway for new types of mobility, such as speed bikes, to manage speed differences among active transport users. 2. Redesign bike lanes to include separate lanes for electric bikes and slower (non-electric) bikes, providing distinct spaces for each type of cyclist. |
| 27 | Transport | Improve coordination among different shared mobility providers and establish national schemes (e.g., unified subscriptions) to enhance user-friendliness. For example:   1. Integrate services from various bike-sharing providers (such as Tier, OV-fiets, etc.) through a single joint subscription; 2. Link car parking tickets to complimentary (free) access to shared bike systems; 3. Develop extensive park-and-ride (P&R) hubs on the outskirts of cities. |
| 28 | Community | Provide municipalities with grants that serve as financial incentives to make spaces and facilities available, such as sports complexes, gymnasiums, schoolyards, and other potential areas for PA. |
| 29 | Sport and Recreation for All | Establish procedures to minimize lobbying, including sports washing, by, for example, setting requirements for sponsor types and sponsorship materials. This should address lobbying from industries such as the automotive sector (which promotes passive mobility), as well as gambling companies and providers of unhealthy foods. |
| 30 | Urban design | Encourage the development of bicycle parking facilities in neighbourhoods by ensuring that homes and streets feature bike parking at the front, visible to residents. These facilities should be easily accessible, provide secure parking, and thus promote cycling. |
| 31 | Healthcare | Provide financial support to physiotherapists for referring patients to regular PA programs after treatment by including these services in the basic insurance. |
| 32 | Workplace | Incorporate regulations into the 'Building Works Decree for the Living Environment' (Besluit Bouwwerken Leefomgeving) to promote PA in new buildings and office spaces, such as positioning stairs as a central feature rather than relying solely on elevators. |
| 33 | Education | Implement measures to reduce nuisance and vandalism in schoolyards to ensure they can be safely opened. For instance, provide subsidies for enforcement around schoolyards, install camera surveillance, and improve lighting. |
| 34 | Workplace | Discontinue reimbursement for car use and car lease schemes for commuting. |
| 35 | Workplace | Include and prioritize PA in the risk assessment and evaluation* process for companies.  *The Risk Inventory and Evaluation (RI&E) forms the foundation of every company's health and safety policy (arbobeleid), helping to identify risks and implement appropriate measures. |
| 36 | Workplace | Mandate minimum facilities to promote PA in medium and large companies, including showers, bicycle parking, and lockers. |
| Recommendations for infrastructure support action | | |
| No. | Domain | Recommendation |
| 1 | Leadership | Implement structural PA policy (and associated PA goals) that extends beyond a usual 4-year government term, ensuring long-term commitment. |
| 2 | Funding and resources | Increase funding for prevention initiatives, with a significant focus on cross-domain PA. |
| 3 | Leadership | Develop PA guidelines (potentially on the basis of the WHO PA guidelines) tailored for vulnerable groups (such as the chronically ill, pregnant women and people with disabilities), to complement the guidance provided by Knowledge Centre for Sport & PA. |
| 4 | Governance | Promote/facilitate the implementation of toolboxes and toolkits developed by Knowledge Centre for Sport & PA and the National Institute for Public Health and the Environment, aimed at securing and utilizing knowledge (including elements of proven effective interventions) in the development of (local) PA policy. This will aid in the effective use of available knowledge.   1. As a condition for subsidies for scientific research, require collaboration with a semi-governmental organization (such as Knowledge Centre for Sport & PA or the National Institute for Public Health and the Environment) to ensure practical translation. |
| 5 | Governance | Launch media campaigns and additional communication strategies to increase awareness and the importance of PA, with a specific focus on the PA guidelines, amongst the general population. |
| 6 | Platforms for interaction | Ensure that PA is a recurring agenda item in the existing 'Impact on Health' (Impact op Gezondheid) steering committee, which operates across ministries. |
| 7 | Leadership | Ensure that PA is recognized as a health-promoting and protective measure within the Public Health Act (Wet Publieke Gezondheid) until a cross-domain PA Act is established. |
| 8 | Governance | Establish cross-domain performance agreements (Key Performance Indicators, or KPIs) focusing on PA for municipalities. These agreements should include interim measurements and hold municipalities accountable, supplementing the national PA goal of having at least 75 percent of the Dutch population meet the PA Guidelines by 2040. This ensures continuity and long-term alignment. Consider including metrics such as a specific number of safe cycle paths and open schoolyards in each neighbourhood. |
| 9 | Governance | Establish procedures and allocate a budget for an effective Plan-Do-Check-Act cycle for 'learning policies,' which should include:   1. Utilization of proven effective elements, including elements of interventions from the RIVM database and international evidence; 2. Integration of insights from monitoring and evaluating policies into the development and adjustment of new and existing policies; 3. Involvement of a researcher as a permanent stakeholder. |
| 10 | Funding and resources | Increase taxes related to products and services that encourage physical inactivity, including taxes on cars (autotaks), and use the revenues from this to encourage PA. |
| 11 | Leadership | Introduce a Sport Act, either alongside or as part of the PA Act, which includes:   1. Designating sport as a public facility, making local governments jointly responsible for organizing and funding sports, thus ensuring better accessibility to PA and sport. This will provide clarity for citizens, associations, and companies on what they can hold governments accountable for. 2. Clearly defining the division of responsibilities between municipalities, provinces, and the national government. |
| 12 | Funding and resources | Reach an agreement with all relevant ministries on allocating a portion of their budgets specifically for PA, proportional to their role in promoting it. This allocation should be explicitly designated as 'budget for PA’ in the national budgets, including those of the Ministry of Health, Ministry of Infrastructure, Ministry of Education, Culture and Science, Ministry of the Interior and Kingdom Relations, and Ministry of Social Affairs and Employment. |
| 13 | Health in all policies | Formulate non-exclusive guidelines for the implementation of national government policies focused on PA, including that national Action Programs should be signed and endorsed by several ministries, and ideally be drafted in co-creation. This universal guideline (for all relevant ministries) should include a check to ensure that the added value of other policy areas is inventoried (this can go beyond just the topic of ‘PA’). Permanent contact persons should be designated within ministries that guarantee this process. |
| 14 | Leadership | Appoint a Secretary of State for PA. |
| 15 | Monitoring | Adopt, recommend, and encourage the use of standardized measures to assess and monitor the local PA environment, including a comprehensive walkability index, with nationwide coverage. |
| 16 | Workforce Development | Ensure the qualification of PA professionals, including community sports coaches (buurtsportcoaches), by establishing a national quality register for all PA professionals. |
| 17 | Workforce Development | Address the underlying reasons for the lack of physical education taught by specialized PA teachers, such as high workloads, budget constraints, and other relevant factors. |
| 18 | Workforce Development | (Re)formulate the conditions for community sports coaches (buurtsportcoaches) with a long-term perspective:   1. Revise and narrow the scope of tasks for community sports coaches. In other words, specify the profiles that have been established for the coaches. 2. Increase funding (e.g., Brede Regeling Combinatiefuncties) for community sports coaches, as the current 40% is insufficient, and offer long-term contracts to ensure stability. 3. Improve working conditions for community sports coaches, potentially through a collective labour agreement, to reduce turnover, enhance quality, and encourage referrals from healthcare professionals. |
| 19 | Funding and resources | Ensure a clear distinction and better balance in government budgets between PA in (professional) sports and cross-domain ('non-sport') PA. Budget allocations should increasingly emphasize cross-domain PA. |
| 20 | Workforce Development | Ensure the facilitation and funding of the Human Capital Agenda* to stimulate dialogue between employers and employees on labor market development and to encourage them to make commitments.  *The Human Capital Agenda (HCA) identifies the bottlenecks and solutions within the sports sector, encompassing both professionals and volunteers. |
| 21 | Governance | Establish procedures to regulate the influence of private parties on policy development related to PA, such as the influence of health insurance companies and the car industry. |
| 22 | Monitoring and Intelligence | Allocate a budget for improved monitoring of PA. For instance, use objective measurement methods, such as activity trackers, to monitor PA on a national level. |
| 23 | Workforce Development | Improve working conditions for all PA professionals, including a salary increase to prevent the outflow of talent to other sectors. |
| 24 | Monitoring and Intelligence | Utilize GPS systems of electric bikes (including shared bikes) to monitor bottlenecks for cyclists, such as areas where speeds decrease. |
| 25 | Platforms for interaction | Create positions where policymakers are affiliated with multiple ministries (e.g., a policymaker from the Ministry of Health also being contracted to the Ministry of Infrastructure and Water Management). This ensures that policymakers have shared interests, facilitates cross-domain collaboration, and more strongly integrates PA across various ministries. |
| 26 | Platforms for interaction | Ensure the safeguarding of overarching bodies that focus on interactions between the government and civil society, while strengthening the role of relevant ministries within these bodies. |

*^1^ With STOMP, priority is given to sustainable modes of transport in the design process, whilst less priority is given to less sustainable modes. The central order of priority is as follows: walking, cycling, public transport, mobility as a service and personal transport.*
